# Reducing Fish Meal Dependency in Juvenile Yellowtail Diets Using Composite By-Product Protein Mixtures

**DOI:** 10.3390/ani16071029

**Published:** 2026-03-27

**Authors:** Amal Biswas, Ryoma Maruyama, Hiroshi Fushimi, Hiroya Sato, Hideki Tanaka

**Affiliations:** 1 Aquaculture Research Institute, Kindai University, Uragami, Wakayama 649-5145, Japan; 2 RegenWorks Co., Ltd., Iwayado, Odaka, Minamisoma 979-2157, Fukushima, Japan

**Keywords:** yellowtail, shark by-product meal, surimi by-product, miscellaneous by-product, fish meal replacement, growth

## Abstract

A 6-week feeding trial evaluated composite protein mixtures as partial replacements for fish meal (FM) in diets for juvenile yellowtail (*Seriola quinqueradiata*). Five diets were prepared: a control diet (C) with FM as the main protein source, and four test diets in which 25% and 35% of FM protein were replaced by either a composite mixture of SSM (44% shark by-product meal + 56% surimi by-product mixture; SS25 and SS35) or SMM (58% shark by-product meal + 42% other by-product mixture; SM25 and SM35). Fish (initial mean weight 0.85 g) were stocked at 30 fish per 500-L tank, with three replicate tanks per treatment. Growth performance indicators, including final mean weight, percent weight gain, specific growth rate, daily feeding rate, and survival, did not differ significantly among treatments (*p* > 0.05). Feed efficiency was significantly higher in SS25 and SS35 than in the control (*p* < 0.05), whereas no significant differences were observed among the by-product-based diets. Whole-body proximate composition was unaffected, except for crude ash. The levels of key n-3 fatty acids, eicosapentaenoic acid and docosahexaenoic acid, were significantly lower in SM25 and SM35 than in the control (*p* < 0.05). These results indicate that up to 35% of FM protein can be replaced with shark by-product-based mixtures without compromising growth in juvenile yellowtail.

## Simple Summary

The increasing cost and limited availability of fish meal (FM), driven by stagnant global production and rising demand from aquaculture, have intensified the need for alternative protein sources in aquaculture feeds. One promising approach is to combine nutritionally complementary by-products rather than relying on a single ingredient. Shark by-product meal is rich in protein, while other food industry residues, such as surimi by-product and miscellaneous by-product, contain moderate levels of nutrients but remain underutilized. In this study, juvenile yellowtail (*Seriola quinqueradiata*) were fed diets in which 25% or 35% of FM protein was replaced with composite mixtures of shark by-product meal combined with either surimi by-product or miscellaneous by-product. After a 6-week feeding period with three replicate tanks per treatment, feed efficiency increased while growth performance and survival were not negatively affected by FM replacement. These results suggest that blending high- and moderate-protein by-products can effectively reduce FM use without compromising growth in juvenile yellowtail, supporting more sustainable feed development for marine aquaculture.

## 1. Introduction

Fish meal (FM) has traditionally been the primary dietary protein source in aquafeeds due to its high crude protein content, balanced amino acid (AA) profile, excellent digestibility, and palatability. However, global increases in aquafeed demand, limited wild fish resources, rising FM costs, and sustainability concerns have created a strong incentive to identify alternative protein sources for aquaculture feeds [1]. Consequently, the development and evaluation of nutritionally viable, economically feasible, and environmentally sustainable FM replacers have become major research priorities worldwide.

A wide range of ingredients have been investigated as alternatives to FM for aquafeed applications, with studies focusing on AA composition, digestibility, palatability, growth performance, and health implications across various cultured species [2]. In general, plant protein sources have received considerable attention due to their abundant supply and relatively low cost [3,4,5,6]. However, the poor nutritional quality of some plant ingredients, together with the presence of anti-nutritional factors (ANFs), limits their application, particularly in carnivorous fish species [5,6,7]. High levels of FM replacement using a single alternative protein source may suppress fish growth [8,9], whereas combining multiple protein sources can improve dietary nutritional quality by more effectively meeting AA requirements and enhancing the potential for FM replacement. For example, diets based solely on plant protein meals may negatively affect fish growth [10], whereas combinations of multiple plant protein sources designed to minimize AA deficiencies can support satisfactory growth performance [3,11]. Moreover, improved growth performance has been reported when FM was replaced by composite mixtures of terrestrial, plant, or rendered animal proteins [12,13,14,15,16] or with blends of animal proteins [17,18]. These findings provide a strong basis for the hypothesis that combining nutritionally complementary ingredients can enhance diet formulation and expand the applicability of alternative protein sources in carnivorous fish species.

Despite extensive research on alternative protein sources for aquafeeds, several by-products with potential nutritional value remain underutilized in aquaculture. Many of these by-products have traditionally been used in livestock feeds or discarded as organic fertilizers, primarily due to their relatively low crude protein content or imbalanced AA profiles when considered as a sole protein source. Nevertheless, such materials may represent valuable feed resources when strategically combined with high-protein ingredients to complement their nutritional deficiencies. Among animal-derived by-products, shark by-product meal (SM) has attracted attention because of its high crude protein content, favorable AA composition, and good digestibility. Previous studies have demonstrated that up to 75% of FM protein can be replaced by SM without compromising growth performance in red sea bream (*Pagrus major*) [19] and yellowtail (*Seriola quinqueradiata*) [20]. The SM used in this and previous studies [19,20] was prepared from bycatch. Although the Inter-American Tropical Tuna Commission (IATTC) has set a catch limit for sharks at 39,000 tons under its resolution [21], the total blue shark bycatch in Japan has been approximately 6000–7000 tons in recent years [22], suggesting that blue shark stocks are not being overfished and that Japan is not violating international regulations.

Some low-protein by-products, such as surimi by-products and other miscellaneous by-products, are still either overlooked or underestimated as feed ingredients. Surimi by-products are generated during the manufacturing process and also include products that cannot be marketed due to not meeting specifications after processing. These residues are generally used as feed for livestock, such as chickens and pigs, or are disposed of by industrial waste processors. Since SM has a high protein content, combining it with low-protein by-products, such as surimi by-product meal (SBPM) or other miscellaneous low-protein-value by-products (MBPM) derived from the food industry, may provide an effective approach to upgrading the nutritional value of these underutilized materials and expanding their applicability in aquafeeds. However, information on the use of such composite mixtures, particularly for carnivorous fish species, remains extremely limited.

Yellowtail (*S. quinqueradiata*) is one of the most economically important marine carnivorous fish species in Japan, accounting for the highest production volume among marine finfish in the aquaculture sector and sustaining strong consumer demand [23]. Previous studies have investigated the replacement of FM with various alternative ingredients in yellowtail diets, including plant protein sources, terrestrial animal by-products, and other marine-derived meals, with varying degrees of success depending on inclusion levels and nutritional supplementation strategies [19,24,25,26,27,28,29]. However, no studies have evaluated the nutritional efficacy of composite diets combining SM with low-protein fishery by-products, such as SBPM and MBPM, in yellowtail.

Therefore, the present study aimed to evaluate the feasibility of replacing FM with blended protein sources consisting of SM combined with SBPM or MBPM in the diets of juvenile yellowtail. Specifically, this study assessed the effects of graded FM replacement levels by using these composite mixtures on growth performance, feed utilization, and body composition. The findings are expected to provide new insights into the effective utilization of underexploited fishery by-products and contribute to the development of more sustainable and cost-effective aquafeeds for carnivorous marine species.

## 2. Materials and Methods

### 2.1. Ethical Statement

The present study was reviewed and approved by the Aquaculture Research Institute, Kindai University, Japan (approval number: ARIKU-AEC-2025-10). To minimize fish suffering, all necessary measures were properly implemented, including the use of anesthesia and fasting before and after measurements.

### 2.2. Processing of Products and Nutritional Comparison

In this study, three by-products (SM, SBPM, and MBPM) were used, all provided by RegenWorks Co., Ltd. (Minamisoma, Japan). As described by Biswas et al. [19], SM was prepared using fresh fillets of blue shark (*Prionace glauca*) after removing the skin, fins, and internal organs. The shark meat was dehydrated to a moisture content below 5% using a vacuum separator (FED-1000, F·E·C Co., Ltd., Aioi, Japan) at low temperature (<50 °C) and subsequently pulverized with a feather mill (YTK311K, Yutaka Manufacturing Co., Ltd., Tokyo, Japan) to achieve a particle size of 300–600 µm. SBPM was produced from by-products collected exclusively from the surimi industry, excluding other sources. In contrast, MBPM included processed surimi-based products such as kamaboko (fish cake), tempura (e.g., burdock and vegetable tempura), and other similar items. However, MBPM did not contain terrestrial meat. After collection, the raw materials for both SBPM and MBPM were subjected to vacuum drying and grinding following the same procedure used for SM preparation. All products were stored in a freezer (approximately 4 °C) until use. The composite mixtures were adjusted as follows: SM 44% + SBPM 56% (SSM) and SM 58% + MBPM 42% (SMM); this allowed us to maintain a protein content of approximately 70%, comparable to that of the FM used in this study. These composite mixtures were used to replace FM protein.

Proximate composition, AA composition, free AA composition, and fatty acid (FA) composition of the ingredients are presented in Table 1, Table 2, Table 3 and Table 4, respectively. Although there was marginal variation in crude protein content among the ingredients, crude lipid, ash, and phosphorus contents were remarkably higher in FM (Table 1). All essential AAs (EAAs), except histidine, were higher in both composite mixtures than in FM (Table 2). Similarly, the levels of most of the non-essential AAs (NEAAs) were higher in both composite mixtures compared to FM (Table 2). However, the levels of most free EAAs were remarkably higher in FM than in both test ingredients (Table 3).

Regarding FA composition, while myristic acid (C14:0), palmitic acid (PA, C16:0), palmitoleic acid (C16:1), eicosapentaenoic acid (EPA, C20:5n-3), and docosahexaenoic acid (DHA, C22:6n-3) were higher in FM, oleic acid (OA, C18:1n-9), linoleic acid (LA, C18:2n-6), and α-linolenic acid (LNA, C18:3n-3) were higher in both composite mixtures (Table 4).

### 2.3. Feed Formula and Proximate Composition

Table 5 represents the dietary formula and proximate composition of the diets. Five diets were formulated: FM was the main protein source in the control diet (C), and FM protein in diet C was replaced with SSM and SMM at levels of 25% and 35%; the diets are referred to as SS25, SS35, SM25, and SM35, respectively (Table 5). All ingredients were thoroughly mixed, and a dough was prepared by adding water. The dough was then pelletized using a laboratory pellet machine (12VR-750SDX, Alpha Royal Co., Ltd.; Osaka, Japan). The pellet diameter ranged from approximately 1.2 to 1.9 mm. After low-temperature oven drying (60 °C for 24 h), diets were stored at −20 °C until use.

The experimental diets were isonitrogenous and isolipidic, with no major variations in crude protein or crude lipid contents, which ranged from 53.6% to 54.09% and from 14.2% to 14.6%, respectively (Table 5).

The dietary compositions of EAAs and NEAAs are shown in Table 6. Since the AA composition was generally higher in test ingredients, EAA and NEAA composition showed an increasing trend in diets containing composite mixtures. The composition of free AAs in the experimental diets also resembled the composition of the ingredients (Table 7).

Table 8 shows the FA compositions of the experimental diets. Compared with the control diet, the test diets showed decreased levels of total saturated FAs and total n-3 polyunsaturated FAs (PUFAs). In contrast, total monounsaturated FAs and n-6 PUFAs were increased in the test diets. The proportions of both EPA and DHA showed a gradual decreasing trend in the test diets.

### 2.4. Fish, Husbandry, and Sampling

After buying yellowtail juveniles from A-marine Kindai (Shirahama, Japan), juveniles were stocked in a 1500 L circular indoor rearing tank at the Aquaculture Research Institute, Uragami Station, Kindai University. Yellowtail juveniles were fed a commercial diet (crude protein 52%, crude lipid 12%; Nisshin Marubeni Feed Co., Ltd.; Tokyo, Japan) to apparent satiation during acclimation period.

Fish were anesthetized with 250 ppm phenoxyethanol (Wako Pure Chemical Industries Ltd.; Osaka, Japan) at the end of the acclimation period, and a group of 30 juveniles with a mean initial weight of approximately 0.85 g were distributed into each 500 L flow-through tank. Each dietary treatment was assigned to three replicate tanks (*n* = 3). During the rearing trial, juveniles were fed their respective diets twice daily to apparent satiation at 08:30 and 14:30 for 6 weeks. The water flow rate was maintained at 5 L min^−1^, and the mean water temperature and concentration of dissolved oxygen were 20.6 ± 1.9 °C and 6.5 ± 0.3 mg L^−1^, respectively. A 12 h light (07:00–19:00) and 12 h dark photoperiod was maintained. Tanks were cleaned daily, and dead fish were collected and weighed when mortality was found.

During the rearing period, fish were sampled and bulk-weighed at weeks 2, 4, and 6 to monitor growth performance. From each tank at the end of experiment, three fish were separated to determine relative organ weights, and the remaining fish were pooled for the analyses of whole-body proximate composition and FA composition. Fish were stored at −40 °C until subsequent analyses.

### 2.5. Calculation of Growth Parameters

Survival rate, percent weight gain (WG), specific growth rate (SGR), daily feeding rate (DFR), feed efficiency (FE), condition factor (CF), and somatic indices including viscerosomatic index (VSI), hepatosomatic index (HSI), stomach somatic index (SSI), pyloric caeca somatic index (PSI), and intestinal somatic index (ISI) were calculated at the end of rearing trial. In addition, protein retention efficiency (PRE) and lipid retention efficiency (LRE) were determined using the following formulae:

Survival rate (%) = 100 × final number of survived fish/initial fish number

WG (%) = 100 × (final mean weight − initial mean weight)/initial mean weight

DFR (g/100 g fish/day) = 100 × total feed intake/[(mean of initial and final no. of fish × mean of initial and final body weight) × rearing period]

FE (%) = 100 × [total wet weight gain (g)/total dry feed intake (g)]

CF = 1000 × (W/L^3^), where W = wet body weight (g) and L = body length (cm)

VSI, HSI, SSI, PSI and ISI (%) = 100 × [wet weight of viscera, liver, stomach, pyloric caeca and intestine (g)/wet body weight (g)]

PRE or LRE (%) = 100 × [(final fish body protein or lipid − initial fish body protein or lipid)/total protein or lipid intake]

### 2.6. Biochemical Analyses

All nutritional analyses were conducted by the Japan Food Research Laboratory (Osaka, Japan). Proximate composition (moisture, crude protein, crude lipid, and ash) of the experimental diets and whole-body samples (initial and final) was determined according to AOAC methods [29]. FA compositions of diets and whole-body samples were analyzed following the method of Folch et al. [30] using a gas chromatograph (GC4000, GL Science, Tokyo, Japan) equipped with a capillary column (InterCap-Pure-WAX, GL Science, Tokyo, Japan). AA compositions of ingredients and diets were determined by using high-performance liquid chromatography (HPLC; GL7700, GL Science, Tokyo, Japan) according to the method described by Teshima et al. [31].

### 2.7. Statistical Analysis

All data were analyzed using SPSS 29 software (IBM Corp., Armonk, NY, USA) for Windows 11. The normality of the data was confirmed through the Kolmogorov–Smirnov test, and homogeneity of variance was also checked by Levene’s statistic. Differences among dietary treatments were evaluated by one-way analysis of variance (ANOVA). When significant differences were detected, Tukey’s multiple comparison test was applied. Statistical significance was accepted at *p* < 0.05. The results are presented as the mean ± standard deviation (SD).

## 3. Results

The variation in growth performance among dietary treatments is presented in Table 9. Statistical analyses showed that there were no significant differences in final mean weight, survival rate, percent WG, SGR, and DFR among the treatments (*p* > 0.05). Although there was no significant difference in FE among the test diets, FE in the SS25 and SS35 groups was significantly higher than in the diet C (*p* < 0.05).

Table 10 shows the variation in CF and relative organ weights among the treatments. The CF of fish fed the SS25 and SM35 diets was significantly higher than that of the control group (*p* < 0.05). Fish fed the SS25 diet showed a significantly higher SSI than that of diet C (*p* < 0.05).

The final whole-body proximate composition is summarized in Table 11. No significant differences were observed in final whole-body moisture, crude protein, or crude lipid contents among the dietary treatments (*p* > 0.05). However, the final whole-body crude ash content of fish fed the SS35, SM25, and SM35 diets was significantly lower than that of fish fed the C diet (*p* < 0.05).

The initial and final whole-body FA compositions are shown in Table 12. The final whole-body contents of C14:0 and C16:1 in fish fed the SS35, SM25, and SM35 diets were significantly lower than those in fish fed the C diet (*p* < 0.05). Fish fed the C diet showed significantly different final whole-body contents of PA, OA, C20:1n-9, C20:4n-6, LNA, and DHA compared with those fed the other diets (*p* < 0.05). Moreover, the final whole-body contents of LA and EPA in fish fed the SM25 and SM35 diets were significantly different from those in fish fed the C diet (*p* < 0.05). No significant differences were observed in other FAs among treatments (*p* > 0.05).

Table 13 shows the variations in PRE and LRE among the treatments. No significant differences were observed in either parameter among dietary treatments (*p* > 0.05).

## 4. Discussion

The combination of different by-products with SM as a partial replacement of FM in the diet of juvenile yellowtail was investigated in this study, and the results show that 35% of FM protein can be replaced by both composite mixtures (SSM and SMM) without compromising the growth potential of yellowtail juvenile. In a previous study on yellowtail [20], up to 50% of FM protein was successfully replaced by SM without compromising growth performance. In the present study, ingredients with lower nutritional value than SM were included to formulate composite mixtures. It was therefore assumed that the utilization efficiency of these composite mixtures, which contain lower-quality by-products, would be inferior to that of SM used alone as a protein source. Accordingly, the FM protein replacement levels were set at 25% and 35% to determine the optimal inclusion level. In this study, the DFR was not significantly affected by either SSM or SMM. However, FE was either similar to or significantly higher in the test diets, suggesting that the experimental diets were well accepted by yellowtail juvenile.

Usually, a low DFR is one of the main reasons for reduced growth performance when FM is replaced by other protein sources due to lower palatability [15,18,32]. The balance of dietary AAs and free AAs is thought to be related to palatability, DFR, and fish growth [33,34]. While some studies suggest that reduced palatability caused by an imbalance in AAs affects growth [35,36], other studies indicate that growth is reduced due to low DFR even when the AA profile is adequate [28,32]. However, in the present study, all dietary EAAs and NEAAs, except histidine, were either similar to or higher than those in the control diet. Although the content of free glutamic acid, known as an umami component, was higher in the test diets, most other free EAAs and NEAAs were lower compared to the control diet. Nevertheless, the lack of a significant difference in DFR suggests that other free AA levels did not negatively affect feed acceptance, thereby supporting good growth in the test diets. In addition, FE in all test diets was either similar to or higher than that in the control diet. Although the lack of digestibility measurements should be considered a limitation, the higher FE suggests that the test diets were effectively digested and assimilated into the fish body to support growth. In a previous study in which SM alone was used to replace FM, neither DFR nor FE was affected in juvenile yellowtail until 50% of FM protein was replaced by SM [20]. While low-FM diets containing plant protein sources have been reported to reduce FE [3,4,37], the relatively higher FE observed in this study may be attributed to the absence of plant proteins in the diets, the sufficient FM levels in the test diets to maintain FE, comparable or improved AA profiles relative to the control diet, or the higher digestibility of the by-products used compared with plant protein sources. The present study further suggests that combining an ingredient of low nutritional value with SM did not negatively affect either DFR or FE in juvenile yellowtail until 35% replacement of FM protein. However, further studies are needed to determine whether long-term rearing can sustain the higher FE observed in by-product-based diets.

Regarding the FA composition of the ingredients, the levels of OA and LA were markedly higher, whereas those of EPA and DHA were lower. However, the total lipid concentrations of both composite mixtures were lower than that of FM; therefore, a comparatively higher amount of fish oil was supplemented in the test diets. Nevertheless, the levels of EPA and DHA remained lower in the test diets than in the control diet. Although limited information is available regarding the FA requirements of juvenile yellowtail, the levels of n-3 PUFA in the test diets were higher than the reported requirement of 2.0–2.4% for yellowtail [38], suggesting that the FA profiles of the test diets were adequate to support good growth in juvenile yellowtail.

It has been demonstrated that, rather than relying on a single ingredient, combining nutritionally complementary by-products represents a promising strategy to enhance diet formulation flexibility and expand ingredient utilization potential. For example, although an all-plant diet significantly reduced growth performance in juvenile bluegill (*Lepomis macrochirus*), a combination of animal- and plant-source proteins produced results comparable to those of the control diet [10]. The combined use of animal- and plant-source proteins has also been shown to successfully replace FM in other species. For instance, a composite mixture of shrimp hydrolysate and plant proteins replaced 22% of FM in largemouth bass (*Micropterus salmoides*) [39]. Similarly, a mixture of meat meal, chicken by-product meal, soy protein concentrate, and corn gluten meal reduced FM inclusion to 40% in diets for red seabream (*Pagrus major*) [15]. On the other hand, combinations of different animal-source proteins have also shown promising results for FM replacement in various species. In Pacific white shrimp (*Litopenaeus vannamei*), a mixture of poultry by-product meal and Antarctic krill meal was concluded to be an excellent substitute for FM [14]. In European seabass (*Dicentrarchus labrax*), 50% of FM was successfully replaced with a mixture of poultry by-product meal and insect exuviae [17]. Likewise, in red seabream, a combination of meat meal and chicken by-product meal successfully replaced 50% of FM [18]. In yellowtail, it has been reported that a mixture of krill meal, soybean meal, corn gluten meal, meat meal, meat and bone meal, poultry feather meal, and blood meal could replace approximately 50% of FM [12]. Furthermore, the present study suggests that a composite mixture of a high-protein ingredient and low-protein by-products can successfully replace up to 35% of FM protein in juvenile yellowtail. As growth performance was similar to or superior to the control diet at replacement levels up to 35%, further investigation is warranted to determine whether higher levels of FM replacement are feasible using this combination in yellowtail.

Although the final whole-body crude lipid content and lipid retention efficiency were not affected by the composite mixtures evaluated in this study, whole-body FA composition was markedly altered. In fish, tissue FA profiles generally reflect dietary FA composition [14,40,41,42] due to the limited capacity of most species to biosynthesize long-chain n-3 PUFA from C18 precursors [43,44]. Accordingly, a significant reduction in whole-body PA was observed in fish fed the test diets compared with the control. From a human nutrition perspective, this shift may be favorable, as excessive PA intake has been associated with dyslipidemia, hyperglycemia and ectopic lipid deposition [45]. In contrast, increased deposition of OA and LA, together with reduced concentrations of EPA and DHA, warrants careful consideration. OA and other C18 monounsaturated FAs are highly digestible and serve as efficient energy substrates in aquaculture diets, supporting growth performance when fish oil is partially replaced [43,46]. In juvenile yellowtail, it has been reported that replacing fish oil with plant oils alters whole-body fatty acid profiles, including reduced EPA and DHA, even when growth performance is maintained [40]. Consistent with this, growth performance and feed utilization were maintained in this study despite the higher dietary inclusion of OA- and LA-rich ingredients. However, elevated dietary OA and LA diluted dietary EPA and DHA, resulting in reduced deposition of these PUFAs in whole-body tissues, a response commonly observed when fish oil is replaced by oleic-rich plant- or animal-derived lipid sources [47]. Although growth was not compromised, the reduction in tissue EPA and DHA may have implications for fish health and product quality. Long-chain n-3 PUFA plays critical roles in maintaining membrane functionality, immune competence and stress resistance in fish [48], while high dietary LA may further influence PUFA metabolism through competitive interactions within FA metabolic pathways [49]. From a consumer standpoint, EPA and DHA are widely recognized as health-promoting FAs with cardioprotective and anti-inflammatory benefits [50]. Therefore, while the present results demonstrate that C18 fatty acids can effectively support growth, maintaining adequate dietary EPA and DHA remains essential to preserve both fish physiological function and the nutritional value of farmed fish as a food source for human consumption. However, although the whole-body EPA and DHA levels in the test groups were significantly lower than those in the control group, they were still higher than the levels reported in the control groups of some species in which FM and fish oil were replaced with alternative sources [18,47].

## 5. Conclusions

The results of this study suggest that either composite mixture can successfully reduce the FM level in the diets of juvenile yellowtail from 70% to 45% (corresponding to 35% replacement of FM protein) without adversely affecting growth performance. The utilization of these discarded food-processing by-products represents a promising strategy to reduce reliance on FM and to support the sustainable development of aquaculture. Although the processing costs of the by-products used in this study have not yet been calculated, their low or negligible cost suggests potential economic benefits. Further studies are warranted to evaluate the applicability of these composite mixtures in the diets of other commercially important species. Moreover, given the still relatively high FM inclusion (45%), further research is warranted to determine the feasibility of more substantial FM reduction using composite mixtures in yellowtail and other aquaculture species.

## Figures and Tables

**Table 1:** Proximate composition (%) of ingredients (on dry basis).

Parameters	Fish Meal	Shark + Surimi By-Product	Shark + Other By-Products
Moisture	5.1	4.6	4.9
Crude protein	69.7	70.0	71.3
Crude lipid	9.2	1.9	6.2
Crude ash	15.2	2.5	1.7
Phosphorus	2.41	0.23	0.24

**Table 2:** Amino acid composition (g/100 g dry basis) of ingredients.

Ingredients	Fish Meal	Shark + Surimi By-Product	Shark + Other By-Products
Essential amino acids (EAAs)			
Arginine	3.61	4.71	4.77
Histidine	2.10	1.66	1.75
Isoleucine	2.57	3.71	3.82
Leucine	4.77	6.18	6.26
Lysine	5.10	6.97	6.89
Methionine	1.78	2.20	2.17
Phenylalanine	2.60	2.92	3.05
Threonine	2.69	3.51	3.6
Tryptophan	0.80	0.93	0.96
Valine	3.17	3.70	3.72
ΣEAA	29.2	36.5	37.0
Non-essential amino acids (NEAAs)			
Alanine	3.57	4.21	4.27
Aspartic acid	5.79	7.45	7.49
Cystine	0.65	0.79	0.81
Glutamic acid	8.11	12.60	12.3
Glycine	4.01	2.84	2.95
Proline	2.51	2.31	2.46
Serine	2.47	3.06	3.07
Tyrosine	2.09	2.61	2.64
ΣNEAA	29.2	35.9	36.0

**Table 3:** Free amino acid composition (mg/100 g dry basis) of ingredients.

Diets	Fish Meal	Shark + Surimi By-Product	Shark + Other By-Products
Essential amino acids (EAAs)			
Arginine	111	1	3
Histidine	651	3	5
Isoleucine	62	4	5
Leucine	131	12	17
Lysine	121	21	30
Methionine	5	7	11
Phenylalanine	57	8	11
Threonine	61	2	2
Tryptophan	12	nd *	nd *
Valine	86	9	13
ΣEAA	1297	67	97
Non-essential amino acids (NEAAs)			
Alanine	210	26	38
Aspartic acid	29	4	6
Cystine	nd *	nd *	nd *
Glutamic acid	112	550	188
Glycine	60	91	51
Proline	57	2	3
Serine	41	1	2
Tyrosine	55	7	9
ΣNEAA	564	681	297

* nd, not detected (detectable range 0.01%).

**Table 4:** Fatty acid composition (% of total fatty acids) of ingredients.

Fatty Acids	Fish Meal	Shark + Surimi By-Product	Shark + Other By-Products
C14:0	6.7	0.8	0.5
C15:0	0.5	0.1	0.0
C16:0 (PA)	19.8	12.3	11.3
C18:0	4.5	4.9	5.4
ΣSFA	31.5	18.1	17.2
C16:1	7.2	1.3	1.0
C18:1n-9 (OA)	10.0	29.3	35.8
C20:1n-9	0.5	1.3	1.1
ΣMUFA	17.7	31.9	37.9
C18:2n-6 (LA)	0.2	14.8	22.4
C20:3n-6	0.1	0.2	0.1
C20:4n-6	1.1	2.4	1.6
C22:5n-6	0.4	0.5	0.3
Σn-6	1.8	17.9	24.4
C18:3n-3 (LNA)	0.8	3.5	5.1
C18:4n-3	1.9	0.2	0.0
C20:4n-3	0.5	0.3	0.1
C20:5n-3 (EPA)	13.7	5.6	2.4
C22:5n-3	1.6	3.1	2.0
C22:6n-3 (DHA)	19.1	14.5	7.6
Σn-3	37.6	27.2	17.2
ΣPUFA	39.4	45.1	41.6

ΣSFAs, total saturated fatty acids; ΣMUFAs, total monounsaturated fatty acids; ΣPUFAs, total polyunsaturated fatty acids.

**Table 5:** Formula and proximate composition of diets used to feed yellowtail.

Ingredients (%)	C	SS25	SS35	SM25	SM35
Fish meal ^1^	70.00	52.50	45.50	52.50	45.50
Shark + surimi by-product	0.00	17.50	24.50	0.00	0.00
Shark + other by-product	0.00	0.00	0.00	17.50	24.50
Fish oil ^2^	8.00	9.40	10.00	8.50	8.60
Wheat flour ^3^	8.00	8.00	8.00	8.00	8.00
α-Starch	8.00	4.00	2.50	5.00	3.50
Vitamin mix ^4^	2.00	2.00	2.00	2.00	2.00
Mineral mix ^4^	2.00	2.00	2.00	2.00	2.00
Stay-C 35	0.20	0.20	0.20	0.20	0.20
Calcium phosphate	0.30	0.40	0.50	0.40	0.50
Taurine	0.24	0.32	0.35	0.30	0.32
Cellulose	1.26	3.68	4.45	3.60	4.88
Proximate composition (%)					
Crude protein	53.6	54.3	54.2	54.2	54.9
Crude lipid	14.6	14.4	14.4	14.2	14.5
Crude ash	14.1	12.2	11.2	12.2	10.7

^1^ TASA, Lima, Peru (crude protein, ca. 70%; crude lipid, ca. 9%); ^2^ Tsuji Oil Co., Ltd. (Tokyo, Japan); ^3^ Nisshin Flour Milling Inc. Tokyo, Japan; ^4^ Marubeni Nisshin Feed Co., Ltd. (Tokyo, Japan) formula.

**Table 6:** Amino acid composition (g/100 g diet, dry basis) of experimental diets.

Diets	C	SS25	SS35	SM25	SM35
Essential amino acids (EAAs)					
Arginine	2.53	2.72	2.80	2.73	2.81
Histidine	1.47	1.39	1.36	1.41	1.38
Isoleucine	1.80	2.00	2.08	2.02	2.11
Leucine	3.34	3.59	3.68	3.60	3.70
Lysine	3.57	3.90	4.03	3.88	4.01
Methionine	1.25	1.32	1.35	1.31	1.34
Phenylalanine	1.82	1.88	1.90	1.90	1.93
Threonine	1.88	2.03	2.08	2.04	2.11
Tryptophan	0.56	0.58	0.59	0.59	0.60
Valine	2.22	2.31	2.35	2.32	2.35
ΣEAA	20.43	21.71	22.22	21.80	22.34
Non-essential amino acids (NEAAs)					
Alanine	2.50	2.61	2.66	2.62	2.67
Aspartic acid	4.05	4.34	4.46	4.35	4.47
Cystine	0.46	0.48	0.49	0.48	0.49
Glutamic acid	5.68	6.46	6.78	6.41	6.70
Glycine	2.81	2.60	2.52	2.62	2.55
Proline	1.76	1.72	1.71	1.75	1.74
Serine	1.73	1.83	1.87	1.83	1.88
Tyrosine	1.46	1.55	1.59	1.56	1.60
ΣNEAA	20.44	21.61	22.07	21.63	22.10

**Table 7:** Free amino acid composition (mg/100 g diet, dry basis) of experimental diets.

Diets	C	SS25	SS35	SM25	SM35
Essential amino acids (EAAs)					
Arginine	78	58	51	59	51
Histidine	456	342	297	343	297
Isoleucine	43	33	29	33	29
Leucine	92	71	63	72	64
Lysine	85	67	60	69	62
Methionine	4	4	4	5	5
Phenylalanine	40	31	28	32	29
Threonine	43	32	28	32	28
Tryptophan	8	nd *	nd *	nd *	nd *
Valine	60	47	41	47	42
ΣEAA	908	686	601	692	608
Non-essential amino acids (NEAAs)					
Alanine	147	115	102	117	105
Aspartic acid	20	16	14	16	15
Cystine	nd *	nd *	nd *	nd *	nd *
Glutamic acid	78	155	186	92	97
Glycine	42	47	50	40	40
Proline	40	30	26	30	27
Serine	29	22	19	22	19
Tyrosine	39	30	27	30	27
ΣNEAA	395	415	423	348	329

* nd, not detected (detectable range 0.01%).

**Table 8:** Fatty acid composition (% of total fatty acids) of experimental diets.

Fatty Acids	C	SS25	SS35	SM25	SM35
C14:0	6.9	5.8	5.6	5.3	4.6
C15:0	0.5	0.4	0.4	0.4	0.3
C16:0 (PA)	20.5	17.8	17.4	17.3	16.1
C18:0	4.7	4.9	5.0	5.1	5.3
ΣSFA	32.6	28.9	28.4	28.1	26.3
C16:1	7.4	6.3	6.0	5.8	5.1
C18:1n-9 (OA)	10.3	11.7	12.2	15.1	16.5
C20:1n-9	0.5	0.5	0.6	0.7	0.8
ΣMUFA	18.3	18.5	18.8	21.6	22.4
C18:2n-6 (LA)	0.2	3.1	4.3	6.1	6.9
C20:4n-6	1.1	1.1	1.1	1.1	1.1
C22:5n-6	0.4	0.4	0.4	0.3	0.3
Σn-6	1.8	4.6	5.8	7.5	8.3
C18:3n-3 (LNA)	0.8	0.9	1.0	1.5	1.8
C18:4n-3	2.0	1.6	1.6	1.5	1.3
C20:4n-3	0.5	0.4	0.4	0.4	0.3
C20:5n-3 (EPA)	14.2	12.1	11.8	11.0	9.8
C22:5n-3	1.7	1.9	2.0	1.8	1.9
C22:6n-3 (DHA)	19.7	17.3	17.0	16.1	14.7
Σn-3	38.9	34.3	33.8	32.4	29.7
ΣPUFA	40.6	38.9	39.6	39.9	38.0

ΣSFAs, total saturated fatty acids; ΣMUFAs, total monounsaturated fatty acids; ΣPUFAs, total polyunsaturated fatty acids.

**Table 9:** Growth performance of yellowtail fed with different diets for 6 weeks.

Parameters	C	SS25	SS35	SM25	SM35	*p*-Value
IMW (g)	0.85 ± 0.00	0.85 ± 0.00	0.85 ± 0.00	0.85 ± 0.00	0.85 ± 0.00	
FMW (g)	11.87 ± 1.41	12.93 ± 1.16	11.62 ± 0.60	11.98 ± 0.58	12.34 ± 0.84	0.539
Survival rate (%)	93.32 ± 3.34	94.41 ± 5.12	92.24 ± 5.11	95.61 ± 5.14	93.34 ± 5.82	0.934
WG (%)	1300.42 ± 166.61	1431.21 ± 137.81	1273.53 ± 69.82	1314.94 ± 68.83	1359.43 ± 99.03	0.518
SGR (%/day)	6.27 ± 0.28	6.49 ± 0.22	6.24 ± 0.12	6.31 ± 0.12	6.38 ± 0.16	0.529
DFR (%)	3.55 ± 0.11	3.42 ± 0.06	3.41 ± 0.11	3.48 ± 0.08	3.41 ± 0.07	0.303
FE (%)	114.32 ± 1.21 ^a^	119.24 ± 1.81 ^b^	119.43 ± 3.02 ^b^	116.81 ± 1.71 ^ab^	118.03 ± 0.62 ^ab^	0.037

IMW, initial mean weight; FMW, final mean weight; WG, weight gain; SGR, specific growth rate; DFR, daily feeding rate; FE, feed efficiency. Values are the mean ± SD of three replicate samples. Means in a row with different superscripts are significantly different (*p* < 0.05, Tukey’s test).

**Table 10:** Biometric indices of yellowtail fed with different diets for 6 weeks.

Indices	C	SS25	SS35	SM25	SM35	*p*-Value
CF	13.14 ± 0.52 ^a^	15.25 ± 1.19 ^b^	14.48 ± 0.70 ^ab^	14.20 ± 0.97 ^ab^	15.57 ± 1.23 ^b^	0.002
VSI	9.15 ± 0.71	9.69 ± 0.74	9.80 ± 0.52	9.21 ± 0.46	9.45 ± 0.12	0.209
HSI	1.45 ± 0.78	1.34 ± 0.37	1.41 ± 0.33	1.33 ± 0.44	1.43 ± 0.30	0.990
PSI	1.59 ± 0.23	1.96 ± 0.21	1.62 ± 0.10	1.67 ± 0.19	1.83 ± 0.21	0.458
SSI	1.08 ± 0.17 ^a^	0.87 ± 0.11 ^b^	1.04 ± 0.27 ^a^	0.92 ± 0.25 ^ab^	0.79 ± 0.20 ^ab^	0.012
ISI	1.78 ± 0.64	2.18 ± 0.68	2.31 ± 0.65	1.91 ± 0.24	2.00 ± 0.22	0.122

CF, condition factor; VSI, viscerosomatic index; HSI, hepatosomatic index; PSI, pyloric caeca somatic index; SSI, stomatosomatic index; ISI, intestinosomatic index. Values are the mean ± SD of three replicate samples. Means in a row with different superscripts are significantly different (*p* < 0.05, Tukey’s test).

**Table 11:** Whole-body proximate composition of yellowtail fed the experimental diets for 6 weeks.

Parameters	Initial	Final					*p*-Value
		C	SS25	SS35	SM25	SM35	
Moisture (%)	81.12	77.61 ± 0.52	77.42 ± 0.31	77.84 ± 0.22	77.32 ± 0.33	77.92 ± 0.41	0.403
Crude protein (%)	14.83	16.61 ± 0.41	16.92 ± 0.31	16.72 ± 0.12	16.94 ± 0.12	16.41 ± 0.12	0.269
Crude lipid (%)	0.92	2.83 ± 0.12	3.02 ± 0.11	2.73 ± 0.22	3.14 ± 0.12	3.02 ± 0.21	0.373
Crude ash (%)	3.11	3.02 ± 0.01 ^a^	2.91 ± 0.01 ^ab^	2.83 ± 0.01 ^bc^	2.92 ± 0.01 ^c^	2.72 ± 0.02 ^d^	<0.001

Values are the mean ± SD of three replicate samples. Means in a row with different superscripts are significantly different (*p* < 0.05, Tukey’s test).

**Table 12:** Fatty acid composition (% of total fatty acids) of whole-body samples at initial and final stages.

Fatty Acids	Initial	C	SS25	SS35	SM25	SM35	*p*-Value
C14:0	1.41	3.30 ± 0.10 ^a^	3.21 ± 0.10 ^a^	2.90 ± 0.10 ^b^	3.01 ± 0.10 ^b^	2.80 ± 0.01 ^b^	<0.001
C15:0	0.32	0.31 ± 0.01	0.30 ± 0.01	0.30 ± 0.01	0.31 ± 0.01	0.30 ± 0.01	0.675
C16:0 (PA)	17.31	14.40 ± 0.10 ^a^	13.81 ± 0.30 ^b^	13.43 ± 0.01 ^bc^	13.60 ± 0.01 ^bc^	13.22 ± 0.14 ^c^	<0.001
C18:0	8.51	5.11 ± 0.10	5.02 ± 0.10	5.20 ± 0.11	5.21 ± 0.10	5.21 ± 0.12	0.445
ΣSFA	27.55	23.12 ± 0.12	22.24 ± 0.34	21.83 ± 0.14	22.13 ± 0.12	21.63 ± 0.15	
C16:1	1.92	5.50 ± 0.21 ^a^	5.50 ± 0.11 ^a^	5.21 ± 0.10 ^b^	5.21 ± 0.10 ^b^	5.01 ± 0.11 ^b^	<0.001
C18:1n-9 (OA)	15.21	15.61 ± 0.11 ^a^	16.82 ± 0.30 ^b^	16.90 ± 0.14 ^b^	17.91 ± 0.11 ^c^	18.82 ± 0.30 ^d^	<0.001
C20:1n-9	1.71	6.61 ± 0.10 ^b^	7.01 ± 0.21 ^a^	7.01 ± 0.11 ^a^	6.40 ± 0.11 ^bc^	6.31 ± 0.11 ^c^	<0.001
ΣMUFA	18.84	27.72 ± 0.24	29.33 ± 0.37	29.12 ± 0.17	29.52 ± 0.15	30.14 ± 0.35	
C18:2n-6 (LA)	3.91	3.30 ± 0.10 ^a^	3.91 ± 0.01 ^a^	4.01 ± 0.10 ^a^	5.40 ± 0.10 ^b^	6.81 ± 1.10 ^c^	<0.001
C20:4n-6	2.31	1.70 ± 0.10 ^a^	1.50 ± 0.11	1.51 ± 0.01 ^b^	1.51 ± 0.01 ^b^	1.50 ± 0.11 ^b^	0.001
C22:5n-6	1.02	0.61 ± 0.11	0.51 ± 0.10	0.50 ± 0.11	0.50 ± 0.01	0.50 ± 0.10	0.227
Σn-6	7.24	5.61 ± 0.14	5.82 ± 0.14	6.02 ± 0.15	7.41 ± 0.13	8.80 ± 0.12	
C18:3n-3 (LNA)	0.40	0.70 ± 0.10 ^a^	0.81 ± 0.11 ^bc^	0.90 ± 0.01 ^c^	1.10 ± 0.01 ^d^	1.21 ± 0.10 ^e^	<0.001
C18:4n-3	0.41	1.31 ± 0.01	1.41 ± 0.10	1.31 ± 0.01	1.21 ± 0.11	1.20 ± 0.11	0.081
C20:4n-3	0.30	0.61 ± 0.01	0.70 ± 0.01	0.70 ± 0.11	0.60 ± 0.01	0.60 ± 0.01	0.112
C20:5n-3 (EPA)	8.00	9.60 ± 0.11 ^a^	9.80 ± 0.31 ^a^	9.71 ± 0.10 ^a^	9.11 ± 0.10 ^b^	8.91 ± 0.1 ^b^	<0.001
C22:5n-3	2.51	2.60 ± 0.32	2.90 ± 0.11	2.91 ± 0.11	2.80 ± 0.10	2.80 ± 0.10	0.097
C22:6n-3 (DHA)	26.00	15.81 ± 0.61 ^a^	14.11 ± 0.64 ^b^	14.60 ± 0.23 ^b^	14.12 ± 0.32 ^b^	13.61 ± 0.42 ^b^	0.005
Σn-3	37.62	30.73 ± 0.74	29.73 ± 0.78	30.03 ± 0.30	28.94 ± 0.37	28.43 ± 0.44	
ΣPUFA	44.86	36.3 ± 0.87	35.5 ± 0.91	36.0 ± 0.41	36.3 ± 0.40	37.2 ± 0.47	

Values are the mean ± SD of three replicate samples. Means in a row with different superscripts are significantly different (*p* < 0.05, Tukey’s test).

**Table 13:** Nutrient retention efficiency in yellowtail fed experimental diets for 6 weeks.

Parameters (%)	C	SS25	SS35	SM25	SM35	*p*-Value
Protein	38.83 ± 1.32	39.92 ± 1.62	39.44 ± 0.91	39.22 ± 0.82	38.84 ± 0.32	0.719
Lipid	25.24 ± 0.71	27.64 ± 1.72	25.42 ± 2.93	28.41 ± 0.84	27.82 ± 2.83	0.245

Values are mean ± SD of three replicate samples.

## Data Availability

The data will be made available from the corresponding author upon reasonable request.
